# SLy1-deficiency results in functional impaired, exhausted and senescent NK cells

**DOI:** 10.3389/fimmu.2026.1836862

**Published:** 2026-06-04

**Authors:** Lisa Rebmann, Victoria Schwenck, Carolin Blumendeller, Isabelle Grund, Jannika Botzenhardt, Sandra Beer-Hammer

**Affiliations:** Department of Pharmacology, Experimental Therapy and Toxicology, Institute of Experimental and Clinical Pharmacology and Pharmacogenomic and ICePhA, University of Tübingen, Tübingen, Germany

**Keywords:** exhaustion, NK cells, p53, ribosome, senescence, SLy1/SASH3

## Abstract

**Introduction:**

SLy1 is an emerging adapter protein, exclusively expressed in lymphocytes. In NK cells it serves as ribosomal stabilizer and plays an important role for their maturation, survival and functionality. SLy1-deficient (SLy1^KO^) NK cells exhibit ribosomal instability, which leads to excessive amounts of free ribosomal proteins followed by an accumulation of p53. However, the characterization of the impairment and the dependence on p53 has not yet been elucidated.

**Objective:**

This study aimed to analyze phenotypical and functional characteristics of SLy1- and p53-deficient NK cells and to understand which impairments depend on both proteins.

**Results:**

We established a SLy1^WT/KO^; p53^WT/KO^ mouse strain and were able to reveal that the reported reduction in viability, cytotoxicity and expression of activating surface receptors in SLy1^KO^ NK cells is mediated by p53. Moreover, we observed that a SLy1^KO^ also leads to decreased NK cell numbers and to increased levels of senescence and exhaustion, independently of p53. Further, we detected elevated protein and mRNA levels of the DNA damage response mediators, which could be responsible for the observed phenotypic alterations.

**Conclusion:**

In brief, we demonstrated that SLy1 is indispensable for adequate numbers of viable, activatable NK cells with an intact cytolytic capacity, and that those phenotypic alterations are p53-mediated. Furthermore, the absence of SLy1 leads to senescence and exhaustion of NK cells in an p53-independant manner. These findings correlate with the recently shown association of human SLy1-mutations with specific types of common variable immunodeficiencies. We therefore strongly recommend testing for mutations in the gene locus, especially in patients with unclear immunodeficiencies.

## Introduction

Natural Killer (NK) cells represent, next to B- and T-cells, an important subtype of lymphocytes. However, in contrast to both other members of the lymphocyte group, the activity of NK cells does not depend on somatic gene rearrangement in order to express specific receptors, but their function is well-orchestrated through a variety of germline-encoded inhibitory and activating receptors (1–3). NK cells display immune cells of the earliest defense, targeting virally infected or tumor cells (4, 5). After activation, they are capable of producing and releasing different cytokines, especially Interferon-γ, as well as different chemokines and growth factors (4, 6).

NK cells are present within most lymphoid and non-lymphoid organs. Larger numbers can especially be found in spleen and lungs (7, 8). Depending on the tissue-residency, their maturation, expression of surface receptors and the proliferation capacity varies (9). Based on their expression of CD27 and CD11b, tissue resident NK cells in the lung are categorized as more mature, in comparison to NK cells in other tissues (9–11).

The adapter protein SLy1 (SH3 protein expressed in lymphocytes 1, SASH3) was shown to play an important role for a proper NK cell function (12, 13). The protein is exclusively expressed in lymphocytes and is characterized by a bipartite nuclear localization signal, a SH3 domain and a sterile alpha motif. The human and murine open reading frame share 94% identity in their amino acid sequence and the corresponding gene locus is, in both species, located on the X-chromosome (14). Amino acid 27 of the translated protein displays a serine, which can be phosphorylated by activated serine-kinases (15). Recently, several case studies have reported patients carrying mutations in the *SLy1/SASH3* gene locus, who all display common variable immunodeficiencies. The affected patients show drastic reductions of blood lymphocytes and an increased susceptibility towards infections and autoimmunity (16–19).

To gain more insights about the relationship between SLy1 and a robust immune system, a mouse model with a global SLy1 knockout was established (20). SLy1-deficient (SLy1^KO^) mice are characterized by reduced cell numbers in lymphoid organs such as spleen, thymus and lymph nodes, partially reflecting the clinical findings (20). In particular, the number of NK cells is significantly decreased and SLy1^KO^ mice show an increased susceptibility towards different types of cancer. Furthermore, the surface expression of specific surface receptors was shown to be altered on SLy1^KO^ NK cells (13). A more detailed analysis revealed that an increased amount of free ribosomal proteins is present in the cytoplasm of SLy1^KO^ NK cells and ribosomal proteins co-immunoprecipitated with SLy1, indicating that SLy1 plays a role as ribosomal stabilizer. The presence of the free ribosomal proteins in turn results in an accumulation of p53, which promotes apoptosis and a derailed regulation of genes important for NK cell function (13). Further, an activation of p53 can result in altered cell conditions as apoptosis, senescence or exhaustion (21–24). Senescence is defined as an irreversible cellular state, in which cells no longer progress through the cell cycle, triggered by intrinsic stress responses, as for example DNA damage (25, 26). As a consequence, senescent cells lose their ability to divide, however the cells are still viable (27–29). Senescent immune cells show alterations in their morphology as well as a decreased functionality and their number increases during the process of aging, resulting in inflammation and an imbalanced immune system (24). Cellular exhaustion, however, describes the progressive loss of function as well as the deficiency to proliferate caused by a an extrinsic overstimulation, resulting in a functional impairment (27, 30).

Further, p53 displays the main mediator of DNA damage response (DDR) as double-or single-strand breaks can lead to an activation of the kinases Ataxia telangiectasia and Rad3-related protein (ATR) and Ataxia telangiectasia mutated (ATM) (31–33). In turn, ATM and ATR can activate the Checkpoint kinases 1 and 2 (Chk1 and Chk2), which can further upregulate p53 (33). At the end of the DDR cascade, usually cell cycle arrest, repair processes, or, in the case of severe damage, senescence or apoptosis occur (34).

Previous findings could reveal an interplay between SLy1 and p53. However, the exact mechanism between the proteins in NK cells is, not yet, completely resolved. To gain more understanding about the interaction of both proteins, we established a SLy1-deficent mouse strain with an additional NK cell-specific deletion of p53. We aimed to analyze different phenotypical and functional characteristics of SLy1- and p53- deficient NK cells and evaluate which features are dependent and independent of the presence of both proteins.

## Materials and methods

### Mice

SLy1^KO^ mice were generated by *Reis et al.* (20) based on a C57BL/6N genetic background. SLy1^KO^ mice were further crossed with a floxed p53 mouse strain (B6.129P2-*Trp53*^tm1Brn^/J; strain #008462 Jackson Laboratory). To generate NK cell-specific knockout of p53, mice were further crossed with Ncr1-Cre mice (Ncr1-Cre^+/tg^; provided by V. Sexl (35)) to obtain SLy1^KO^: Ncr1-Cre^+/tg^ animals.

This breeding strategy resulted in four different genotypes. To highlight the respective presence of SLy1 and p53, short terms for each genotype were applied (**Supplementary Table 1**).

Mice were kept in a specific pathogen-free animal facility of the medical faculty of the Eberhard Karls University Tübingen. A twelve-hours day and night rhythm was set up and water and food was freely available at all times. Mice were kept in groups of two to five individuals and were regularly examined by a veterinarian. If accepted by the group, cages were equipped with enrichment options, providing a stimulating and varied surrounding.

Animals used in the context of this work were all aged between eight and 20 weeks and both genders were included. All animal experiments were approved by the local authority and annotated to the Regierungspräsidium Tübingen (AZ 23.5.2013, AZ 26.4.2018 and PH01/23M) and were performed according to the ARRIVE guidelines.

### Preparation of single cells from spleen and lungs

Mice were anesthetized using isoflurane (CP Pharma) and sacrificed by cervical dislocation. Spleen and lung were removed. Spleens were mechanically homogenized using a 70 µm cell strainer (Falcon Thermo Fisher). The obtained single cell suspension was centrifuged (500g, 5 min, 4°C) and erythrocytes were lysed by incubating the cell pellet for three minutes in erythrocyte lysis buffer (0.155 M NH_4_Cl, 0.01 M KHCO_3_ and 0.1 mM EDTA in H_2_Odd) at room temperature. Afterwards, cells were resuspended in cold PBS (Gibco Life Technologies) and were kept on ice for further experiments.

Lungs were cut into small pieces (approx. 2 mm^3^) and were processed using the Lung dissociation kit and the Octo Dissociator with Heaters (both Miltenyi) by strictly following the manufactures’ protocol. Using this protocol, lungs were enzymatically and mechanically dissociated into single cells. Next, erythrocytes were lysed as described above. Afterwards, cells were washed once and were resuspended in PBS.

### Magnetic cell sorting

To obtain isolated NK cells, total splenic cells were counted and NK cells were isolated *via* magnetic cell sorting using the NK Cell Isolation Kit II (Miltenyi) for spleen suspensions. The guidelines provided by the manufacturer were strictly followed. Purity of the isolated cells was verified by random sampling using flow cytometry.

### Cell culture

Lewis Lung carcinoma cells (LLC cells, #CRL-164 ATCC) were cultured in DMEM high glucose medium (Sigma-Aldrich), supplemented with 10% fetal calf serum (FCS), 1% L-Glutamine, 1% Penicillin/Streptomycin (all GE Healthcare) and 0,05 mM β-Mercaptoethanol (Gibco Life Technologies). The cells were passaged and frozen at passage 5 in liquid nitrogen. To thaw the cells, they were warmed in a water bath at 37°C, resuspended in medium and from then on passaged twice a week.

### Killing assay

LLC cells were passaged 24 hours before the start of the experiment. On the day of the experiment, LLC cells were detached from the bottom of the flask (1 ml of trypsin/EDTA (0.05%/0.02%) for 30 seconds, processed into single cell suspension, counted and resuspended in medium to a final concentration of 10^5^ cells/ml. In a 96-well U-bottom plate (Greiner), 10^4^ LLC cells were seeded and incubated for four hours at 37°C, 5% CO_2_ in the presence of 25*10^4^ freshly isolated splenic NK cells. Depending on the number of isolated NK cells duplicates or triplicates per specimen were prepared. After the incubation period, cells were harvested, washed and prepared for flow cytometry analyses (see section “Flow cytometry”). Three different controls were used to ensure correct analysis of the LLC cell viability: 1) LLC cells: alive 2) LLC cells: dead 3) LLC cells: incubated for four hours without NK cells. For the second control, LLC cells were treated with the BD Transcription Factor Buffer Set according to the manufacturer’s instructions. 1 ml Fix/Perm working solution was added to 1x10^6^ LLC cells and incubated at 2-8°C in the dark for 40-50 minutes. Afterwards, cells were washed twice with 2 ml PBS. All samples and controls were then stained with Annexin V and 7-AAD as described below (see section “Flow cytometry”).

### Interleukin-15 stimulation assay

Isolated splenocytes were resuspended in RPMI medium (Sigma-Aldrich) supplemented with 5% FCS, 1% Penicillin/Streptomycin, 1% L-glutamine and 0.1% ß-mercaptoethanol to a final concentration of 10^6^ cells/ml. Cells were stimulated in six well plates with either a low (c = 10 ng/ml) or a high (c = 100 ng/ml) concentration of Interleukin-15 (IL-15, PeptroTech) and unstimulated controls were prepared. Cell suspensions were kept for 48 h or 72 h at 37°C, 5% CO_2_. The 48 h incubation samples were re-stimulated after 24 h, the 72 h specimens after 24 h and 48 h with the respective concentration of IL-15.

After the incubation period, cells were harvested and empty wells were washed once with PBS. Attached cells were removed carefully after 5 min incubating in 2 mM EDTA (Gibco/Thermo Fisher) and gentle detaching using a cell scraper, followed by one more washing step.

### Senescence assay

Senescence of cells was analyzed as previously described (36, 37). In brief, 2x10^6^ single lung or splenic cells were plated into the wells of a 24 well plate and filled up to a final volume of 2 ml with RPMI medium, supplemented with 100 nM Bafilomycin A1 (Cell Signaling Technologies). Cells were incubated for one h at 37°C, 5% CO_2_. Afterwards, 66 µM C_12_FDG (Invitrogen/Thermo Fisher) was added for 2 h. Duplicates per specimen and organ were prepared, as well as one sample without C_12_FDG treatment. Following the incubation period, samples were washed twice with PBS. Specimens were analyzed using flow cytometry.

### Flow cytometry

In all flow cytometry experiments, NK cells were defined as CD45.2^+^ CD3e^-^ CD49b^+^. (**Supplementary Figure 1**) for cell suspension of lung tissue (left) and spleen (right).

All steps, until the sample acquisition, were performed on ice. 10^6^ single cells were transferred into flow cytometry tubes (Sarstedt) and were washed once with cold PBS. Fc receptors were blocked for 15 min with 1 µg/µl anti-CD16/CD32 antibodies (BioLegend). Next, cells were incubated for 25 min in an antibody cocktail containing antibodies against CD45.2, CD3e and CD49b (all BioLegend).

The absolute cell count of NK cells was determined using the following formula:


$$\frac{NK\;cells}{living\;cells\;(flow\;cytometry)}[\%]*living\;cells\;(counted)$$

For the analysis of apoptosis rates cells were resuspended in 100 µl of Annexin V binding buffer and Annexin V was added (both BD Biosciences) in a ratio of 1:200 for 10 min at room temperature. Apoptotic cells were defined as Annexin V^+^.

For the analysis of the NK cell surface markers, one additional antibody (NKG2D, NK1.1, NKp46/NCR1, KLRG1, CD122, CD51, NKG2A, Ly49A, Ly49H or CD94) was added (all BioLegend or BD Biosciences). To distinguish between the CD27^+/-^ and CD11b^+/-^ maturation forms, anti-CD11b and anti-CD27 (both BioLegend) antibodies were added to the antibody cocktail. After the incubation period, cells were washed twice with cold PBS and samples were either analyzed directly or an additional Annexin V staining was performed in order to gate on viable NK cells. The CD45.2^+^ CD3e^-^ CD49b^+^ Annexin V^-^ NK cell population was further analyzed for one of the above-mentioned surface markers, appearing in a bimodal distribution as one positive and one negative population. The positive population was investigated for the frequency of parent and the geometric mean fluorescence intensity.

For the analysis of the LLC cell viability in the killing assay, the collected cells were resuspended in 100 µl of Annexin binding buffer and 0.5 µl Annexin V and 2.5 µl 7-AAD (all BD Biosciences) were added for ten minutes. Afterwards another 100 µl of Annexin binding buffer were added. LLC and NK cells were distinguished based on their size and granularity. LLC cells were further analyzed and dead LLC cells were identified by a high 7-AAD and Annexin V signal (**Supplementary Figure 2**).

The IL-15 stimulated cells were prepared as described above. After staining with the basic surface antibody cocktail and washing, cells were fixed and permeabilized using the eBioscience™ Foxp3/Transcription Factor Staining Buffer Set (Thermo Fisher). To determine the level of proliferation, 5 µl of anti-KI-67 FITC antibody (BD Biosciences) were added for another 30 min, followed by two more washing steps. Cells were resuspended in 300 µl PBS.

To determine the level of senescent cells, cells were prepared as described above and afterwards incubated in the basic antibody cocktail. Then cells were washed and 7-AAD (1:40) was added for 15 min on ice in order to exclude dead cells. Prior to the sample acquisition, 100 µl PBS was added. Gating was performed as follows: firstly, gate was set on single cells, next on the CD45.2^+^ CD3e^−^ CD49b^+^ NK cell population. Dead (7-AAD^+^) NK cells were excluded from this NK cell population. The geometric mean fluorescence intensity (gMFI) of C_12_FDG was then determined and the gMFI of the unstained control was subtracted.

Sample acquisition was performed using a BD Facs Canto II (BD Sciences). Flow cytometry data was analyzed using FlowJo (Version 10).

### p53 signaling pathway array

RNA was extracted from isolated splenic NK cells using the RNeasy Mini Kit (Qiagen). Reverse transcription of RNA was performed with the RT2 First Strand Kit (Qiagen) for transcription of 1-100 ng RNA. Pre-amplification was performed using the RT2 PreAMP PCR Mastermix and the RT2 PreAMP Pathway Primer Mix (both Qiagen). All kits were used according to the provided protocols. To analyze the expression of genes involved in the p53 signaling pathway, the Mouse p53 Signaling Pathway RT2 Profiler PCR Array (Qiagen) was applied and in total the gene expression of 84 relevant genes was analyzed. For the measurement, a LightCycler 480° (Roche) was used. The obtained data set was evaluated using the data analysis web portal geneGlobe (Qiagen). CT values were normalized based on a manual selection of reference genes.

### qPCR

For the qPCR reactions, SYBR Green assays (Qiagen) were used. For each target, specific primers were applied, which were designed using the Universal Probe Library Assay Design Centre (Roche) and are listed in **Supplementary Table 2**. In all reactions, β-actin served as a reference gene and an internal positive control sample was run for each gene. Fold values were calculated for each sample and data was analyzed with the LightCycler 480° (Roche).

### Immunoblotting

Isolated splenic NK cells were lysed in NP-40 lysis buffer (50mM Tris [pH 7.4], 150mM NaCl, 1% NP-40, 2mM EDTA in H_2_Odd), supplemented with 10% protease and 10% phosphatase inhibitor (both Roche). Proteins were afterwards separated based on their molecular weight by SDS-PAGE using 4 – 15% gradient gels (BioRad). After the transfer onto a nitrocellulose membrane (Thermo Fisher), unspecific binding sites were blocked, depending on the primary antibody either in 5% skim milk (Carl Roth) or 5% BSA (Sigma-Aldrich). Membranes were next incubated overnight in the respective primary antibodies, targeting the protein of interest (ATR, Chk1, ATM and Chk2, all Cell Signaling). All antibodies were diluted either in 5% skim milk (Carl Roth) or 5% BSA (Sigma-Aldrich) in TBST.

On the next day, an HRP-labeled secondary antibody, diluted in 5% skim milk or BSA, was added for 1 h at room temperature and after three washing steps the chemiluminescence signal was analyzed using SignalFire (CellSignaling) as substrate and the VersaDoc™ Imaging System (BioRad). β-actin (Abcam) served as reference protein. Blots were evaluated using Image Lab software version 4.0 (Bio-Rad).

### Statistical analysis

Statistical analysis was performed using GraphPad prism (version 9). Comparison between groups was done by unpaired t-tests. A 95% confidence interval was applied in all analyses. Outliers were identified using the ROUT method with Q set to 5%.

## Results

NK cells play an important role in sustaining a fully functional immune system. To provide protection against neoplasia and viral infections, it is essential to have a sufficient number of living, activatable NK cells that are capable of cytotoxic lysis of target cells. To analyze the role of SLy1 during differentiation, function and maturation of NK cells, SLy1^KO^ mice were examined in comparison to SLy1^WT^ controls. The effect of SLy1 deficiency on the number of NK cells, their expression of surface antigens, cytotoxicity, apoptosis, senescence and exhaustion of NK cells was investigated. In particular, we studied which SLy1-mediated effects depend on p53 by analyzing NK cells from double-deficient mice.

### SLy1^KO^ mice show reduced NK cell numbers and an impaired NK cell function

**Figure 1:**
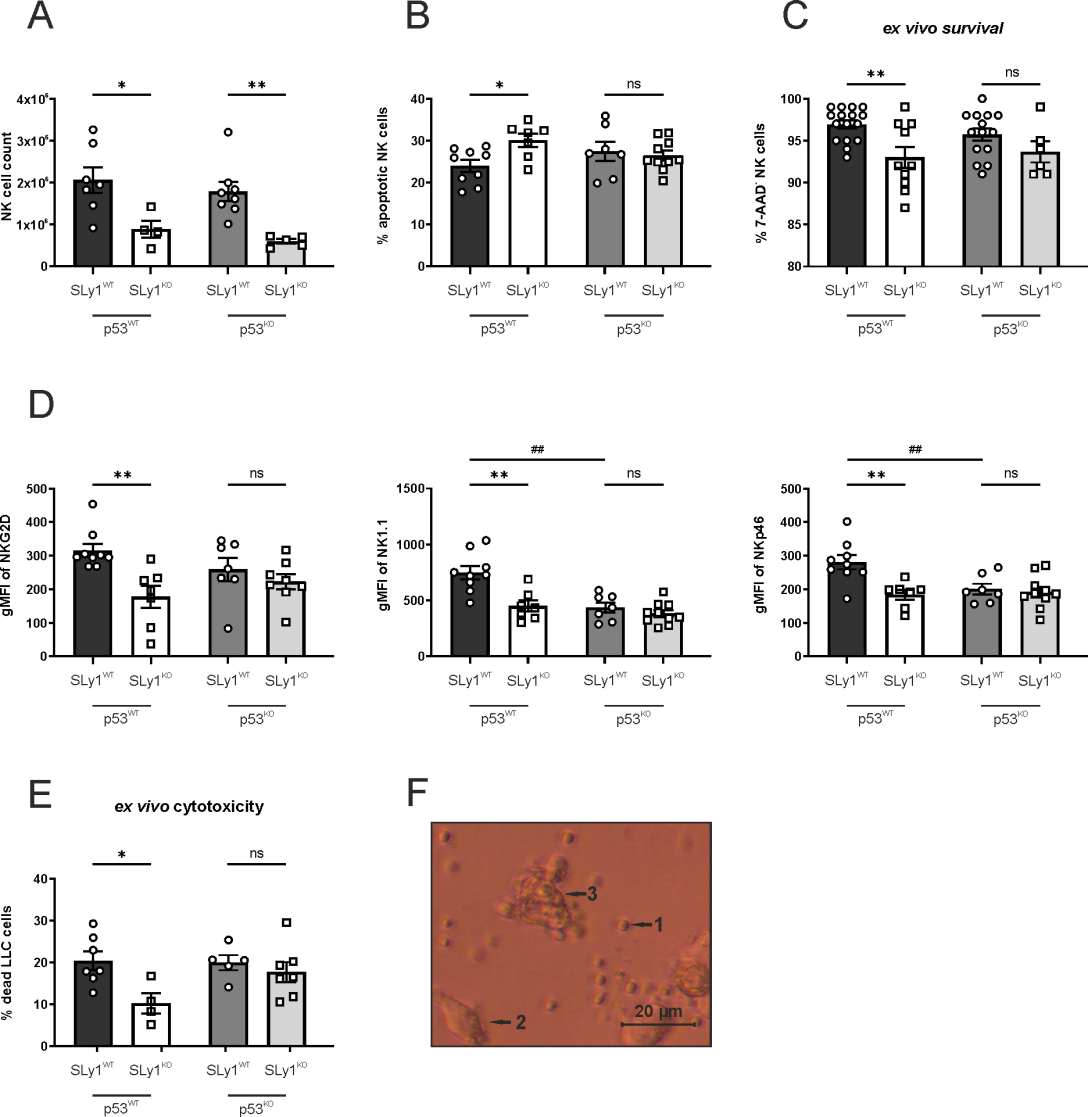
Phenotypic and functional effects of a SLy1 knockout on splenic NK cells in dependence on p53. **(A)** Absolute NK cell count determined *via* flow cytometry in the spleen of mice of the specified genotypes. **(B)** Apoptotic (defined as Annexin V^+^) NK cells. **(C)** Living (defined as 7-AAD^-^) NK cells after four hours under culture conditions. **(D)** Expression of NKG2D, NK1.1 and NKp46 on NK cells. **(E)** *In vitro* cytotoxicity against LLC at a 25:1 effector:target ratio. Dead LLC cells were defined as 7-AAD^+^/Annexin V^+^. In all figures error bars represent standard error of the mean. ∗: p <0.05, ∗∗/##: p <0.01, ns, not significant; unpaired t-test. **(F)** Freshly isolated NK cells (1) attack and lyse LLC cells (2) *in vitro* (see arrow 3). Image was taken after four hours of incubation. Scale bar = 20 µm.

Previous studies have shown that a global deficiency of SLy1 has a negative effect on the number of NK cells in the spleen and lungs. In SLy1^KO^ mice, the percentage of NK cells in ratio to all leukocytes was significantly reduced, while the number of NK cell progenitors remained unaltered (13). These findings were validated in our flow cytometry analyzes and importantly any influence of the floxed *p53* allele could be ruled out. Approximately half as many NK cells were found in the spleen of 20-week-old SLy1^KO^; p53^WT^ mice compared to wildtype control mice of the same age. This effect was still evident in the presence of a simultaneous p53 knockout (**Figure 1A**). Analogously, the absolute number of NK cells in lung tissue was determined. Again, the absolute NK cell count was only about half that of comparable wildtype mice. This effect was still evident in the presence of a simultaneous knockout of p53 (**Supplementary Figure 2A**). The findings suggest that the absence of SLy1 reduces the amount of NK cells in the spleen and lung approximately by half.

Next, the viability of NK cells was examined using flow cytometry. Here, apoptotic NK cells were defined as CD45.2^+^ CD3e^-^ CD49b^+^ Annexin V^+^. Our data analysis showed a significantly higher proportion of apoptotic splenic NK cells in SLy1^KO^; p53^WT^ mice compared to wildtype controls. In contrast, no statistically significant difference was detectable in SLy1^KO^; p53^KO^ mice compared to SLy1^WT^; p53^KO^ controls (**Figure 1B**). In the same manner, pulmonary NK cells were analyzed for apoptosis. Here, no differences were detected between the examined genotypes (data not shown). We conclude that a SLy1 knockout leads to increased apoptosis levels in splenic NK cells and that this effect is p53-mediated.

To assess survivability, the proportion of living (7-AAD^-^) NK cells was determined by flow cytometry after four hours in culture medium. SLy1^KO^; p53^WT^ cells exhibited significantly lower survivability than SLy1^WT^; p53^WT^ NK cells. In contrast, no significant difference was observed between SLy1^KO^; p53^KO^ and SLy1^WT^; p53^KO^ NK cells (**Figure 1C**). Thus, impaired *ex vivo* survivability of SLy1^KO^ NK cells is not dependent on p53.

Further, the expression levels of the activating receptors NKG2D, NK1.1 and NKp46 were quantified using the geometric mean fluorescence intensity (gMFI). We could detect significantly reduced expression levels of all three receptors on splenic SLy1^KO^; p53^WT^ NK cells compared to controls. In contrast, the expression levels of the receptors were not significantly altered on SLy1^KO^; p53^KO^ NK cells compared to SLy1^WT^; p53^KO^ controls (**Figure 1D**). Interestingly, and in contrast to splenic NK cells, pulmonary NK cells did not show any significant differences between the genotypes analyzed in terms of the expression of NKG2D, NK1.1, NKp46 nor in the expression of NKG2A, Ly49A, Ly49H and CD94 (data not shown). Taken together, a SLy1 knockout leads to a p53-mediated reduction in the expression of activating receptors on splenic NK cells but does not affect the receptor expression on pulmonary NK cells.

In order to measure NK cell cytotoxicity, freshly isolated splenic NK cells were incubated for four hours with LLC cells (**Figure 1F**) and afterwards the amount of dead (7-AAD^+^/Annexin V^+^) LLC cells was determined (**Supplementary Figure 2B**). After incubation with SLy1^KO^; p53^WT^ NK cells, only approximately half as many dead LLC cells were detectable as after exposure to SLy1^WT^; p53^WT^ NK cells. LLC cells incubated with SLy1^KO^; p53^KO^ NK or SLy1^WT^; p53^KO^ did not show significant differences of dead cells between both genotypes (**Figure 1E**). Hence, the depletion of SLy1 results in a p53-mediated reduction of NK cell cytotoxicity against LLC cells.

### SLy1-deficiency affects the rate of senescent NK cells

Cellular senescence is a state in which cells are alive but do no longer divide. Typically, cells enter senescence at the end of their lifespan or after irreparable DNA damage has occurred. Phenotypic characteristics of senescent cells include an increase in cell size and granularity, altered gene expression patterns and an elevated β-galactosidase activity in the lysosomal compartment (36, 38, 39). An increase in cell size can be quantified by an elevated forward scatter (FSC) measured by flow cytometry, whereas an increase in granularity leads to a higher side scatter (SSC).

**Figure 2:**
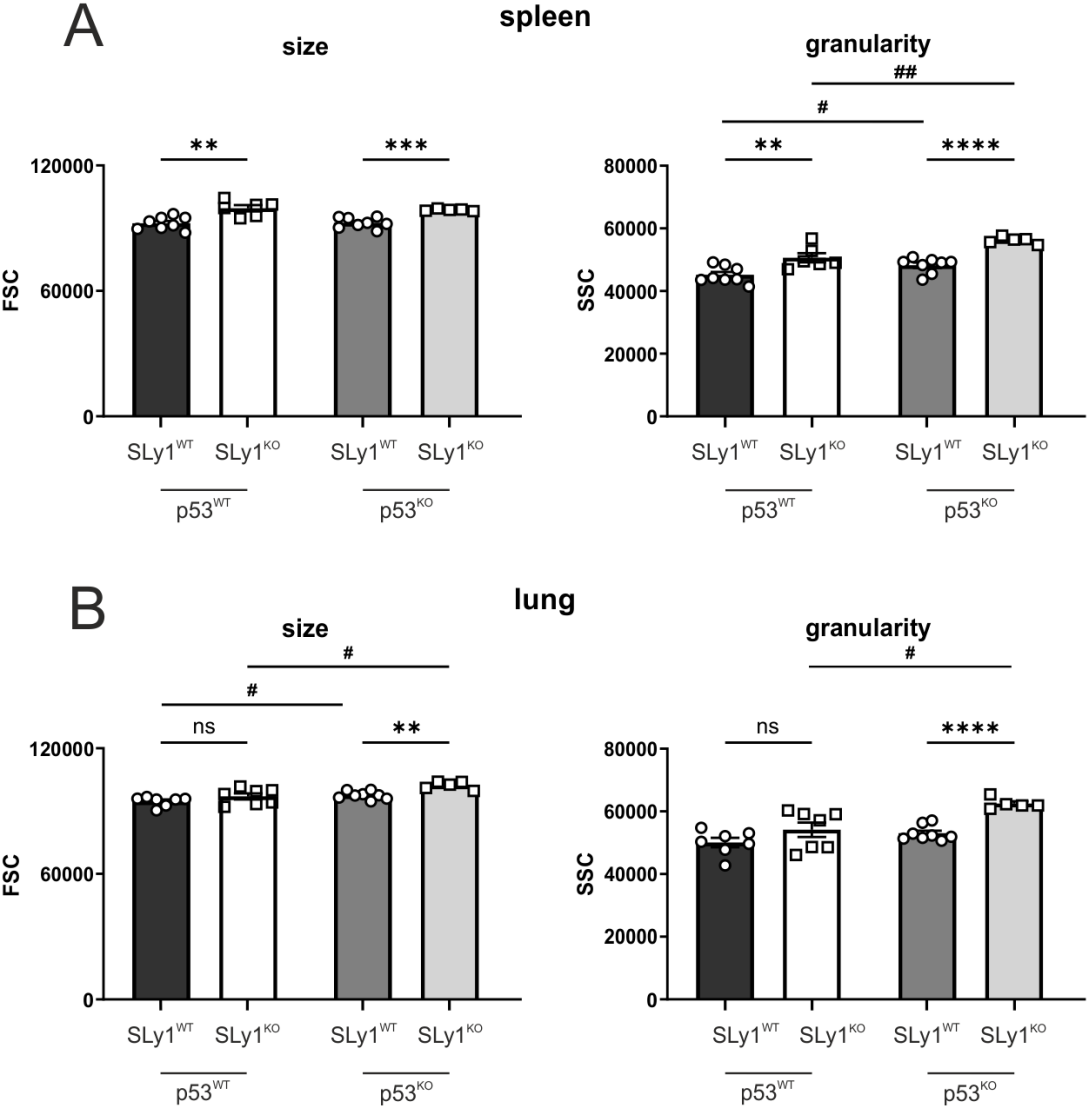
Quantification of cell size and granularity of NK cells of the indicated genotypes for **(A)** spleen and **(B)** lungs. Cell size was measured using FSC. Granularity was measured using SSC. Error bars represent standard error of the mean. ∗/#, p <0.05; ∗∗/##, p <0.01; ∗∗∗, p <0.001; ∗∗∗∗, p <0.0001; ns, not significant; unpaired t-test.

NK cells were isolated from 20-weeks-old SLy1^WT/KO^ or SLy1^WT/KO^; p53^WT/KO^ mice. Splenic SLy1^KO^; p53^WT^ NK cells showed elevated FSC and SSC values compared to wildtype NK cells, indicating a larger cell size and an increased granularity. In the presence of an additional knockout of p53, this effect was still measurable (**Figure 2A****;** **Supplementary Figure 3A**). Pulmonary SLy1^KO^; p53^WT^ NK cells in these mice also showed the same phenotypic alterations towards larger cell size and higher granularity, but the results were not statistically significant due to high fluctuation of the data (**Figure 2B****;** **Supplementary Figure 3B**). However, analysis of FSC and SSC in pulmonary NK cells was repeated in another series of assays (also on 20-week-old mice of the same genotype), where a larger sample size enabled us to reach statistical significance (**Supplementary Figure 3C**). As for the spleen, the increased FSC/SSC values were still detectable in the double deficient SLy1^KO^; p53^KO^ NK cells.

**Figure 3:**
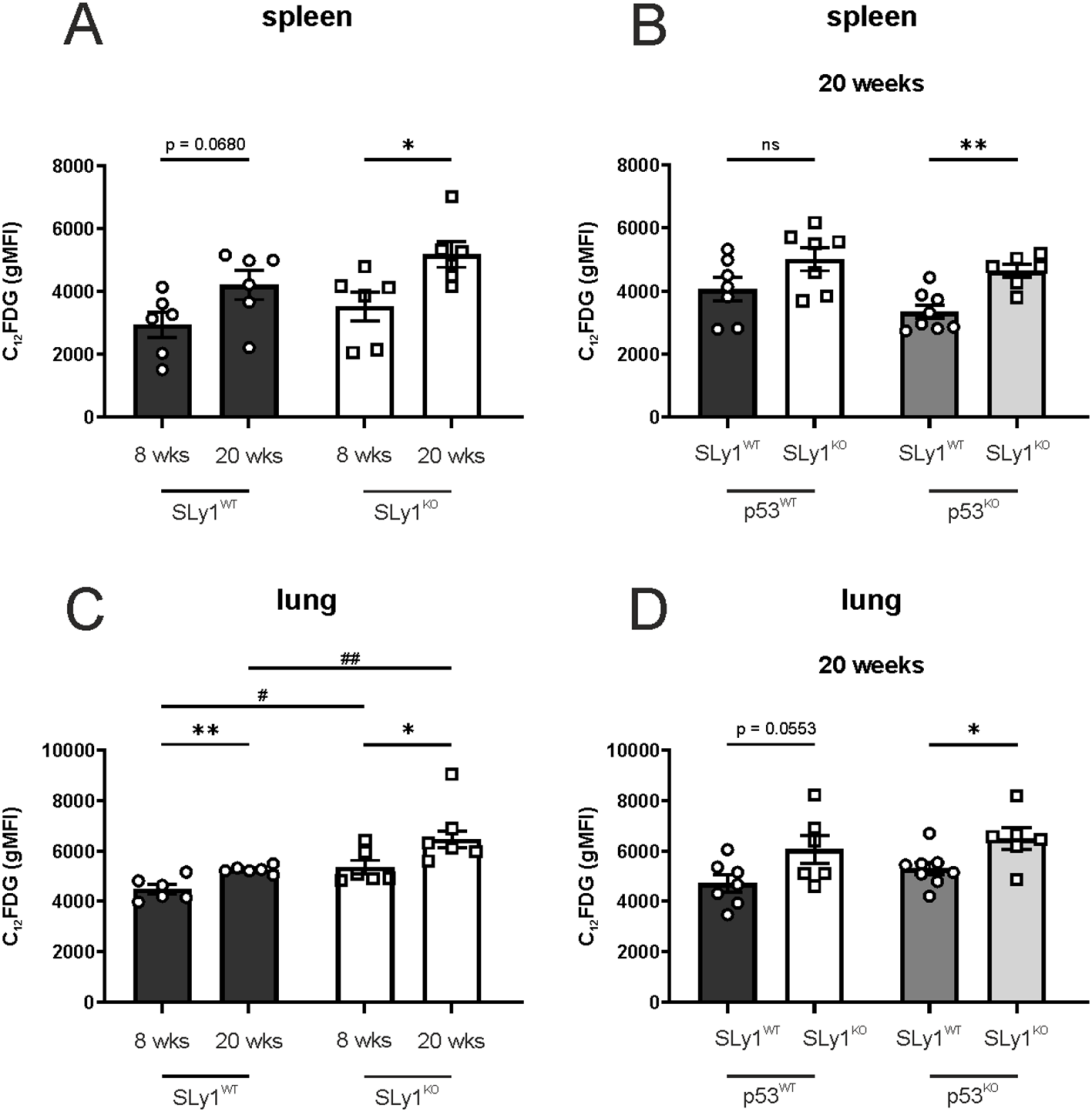
Senescent (C_12_FDG^+^) NK cells in the spleen (upper part) and lungs (lower part). **(A, C)** Comparison of the proportion of senescent NK cells in young (8 weeks) and old (20 weeks) mice depending on SLy1 genotype. **(B, D)** Senescence (in 20-week-old mice) in SLy1 knockout NK cells depending on p53. Error bars represent standard error of the mean. ∗/#, p <0.05; ∗∗/##, p <0.01; ns, not significant; unpaired t-test.

Furthermore, the proportion of senescent NK cells was quantified by analyzing the β-galactosidase activity. C_12_FDG, a substrate of β-galactosidase, is cleaved intracellularly, producing a fluorescent dye, which can be detected using flow cytometry (36, 37). A strong fluorescence intensity indicates high β-galactosidase activity and thus a senescent state of a cell (37). Fluorescence was measured in NK cells obtained from spleen and lungs of the above-mentioned cohort of 20-weeks-old mice. Additionally, the same measurement was performed on NK cells from the spleen and lungs of a much younger cohort of 8-week-old mice, in which we expected the effect of senescence to be not (yet) apparent. In both the spleen (**Figure 3A**) and the lungs (**Figure 3C**), older mice showed increased levels of senescent cells compared to younger mice, regardless of genotype. Although these results are not surprising, they are important to validate the method used.

In order to examine the effect of a SLy1 knockout, mean fluorescent intensity of C_12_FDG was measured in cells from the spleen and lungs of the “older” 20-week-old cohort.

Both splenic (**Figure 3B**) and pulmonary (**Figure 3D**) SLy1^KO^; p53^WT^ NK cells showed a tendency towards increased C_12_FDG values compared to control mice. Although the data was not statistically significant a strong tendency was evident. With an additional NK cell-specific p53 knockout there were still increased senescence rates detectable, suggesting independence of p53. Furthermore, the gMFI of C_12_FDG was compared between the two organs lung and spleen. NK cells in the lungs consistently had a higher C_12_FDG gMFI than splenic NK cells. These results indicate that SLy1^KO^ mice tend to have a greater proportion of senescent NK cells than wildtype mice, older mice have a higher proportion of senescent NK cells than younger mice and the amount of senescent NK cells is larger in the lungs than in the spleen.

### SLy1 knockout influences the rate of exhausted NK cells

Exhaustion describes the loss of function that occurs when a cell enters a state of depletion. In recent decades, knowledge about T cell exhaustion, its clinical relevance and therapeutic reversibility (e.g. by using checkpoint inhibitors) has grown rapidly. NK cell exhaustion has been described in the context of various types of cancer, CMV infection and chronic cytokine stimulation (40, 41). As exhaustion often occurs after chronic NK cell stimulation, it is thought to be a self-regulatory mechanism that prevents an excessive NK cell response (42, 43). NK cells are defined as exhausted, when they display an increased expression of established exhaustion markers along with a dysfunction (38).

According to current knowledge, the detection of the KLRG1 receptor on an NK cell is considered to be an activation marker during acute NK cell activation on the one hand and, on the other hand, a marker for senescent and exhausted NK cells after chronic stimulation (43). KLRG1^+^ NK cells have a more mature phenotype and a lower proliferation capacity than KLRG1^-^ NK cells and it is assumed that NK cells accumulate KLRG1 on their surface during their development (44). Between 30–60% of murine NK cells are positive for KLRG1, depending on tissue-residency and age (10, 45, 46).

**Figure 4:**
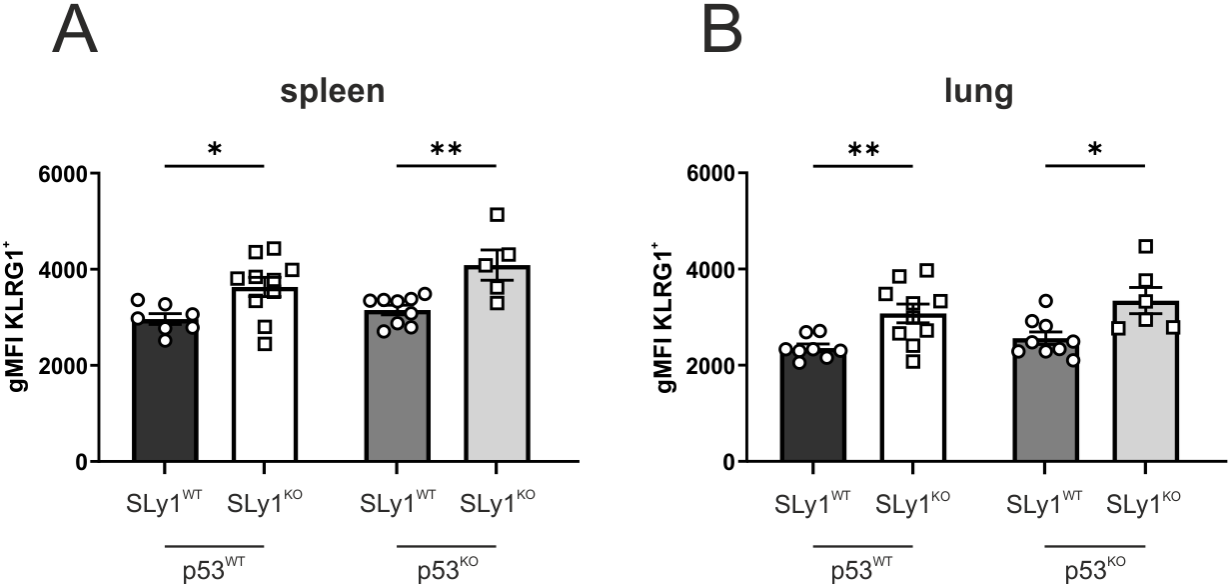
Expression of KLRG1 in dependency of the genotype on **(A)** splenic and **(B)** pulmonary NK cells. Error bars represent standard error of the mean. ∗, p <0.05; ∗∗, p <0.01; unpaired t-test.

We determined the percentage of KLRG1^+^ NK cells as well as the gMFI in the spleen and in the lungs. In wildtype mice, KLRG1 was detected on 27% of splenic and 38% of pulmonary NK cells (data not shown). The measured values approximately represent the range of values published by *Corral et al.* and validate the method used (45). In both organs, the gMFI was significantly elevated in SLy1^KO^; p53^WT^ NK cells depicting an accumulation of KLRG1. This effect was still evident in the presence of a simultaneous p53 knockout (**Figure 4**).

**Figure 5:**
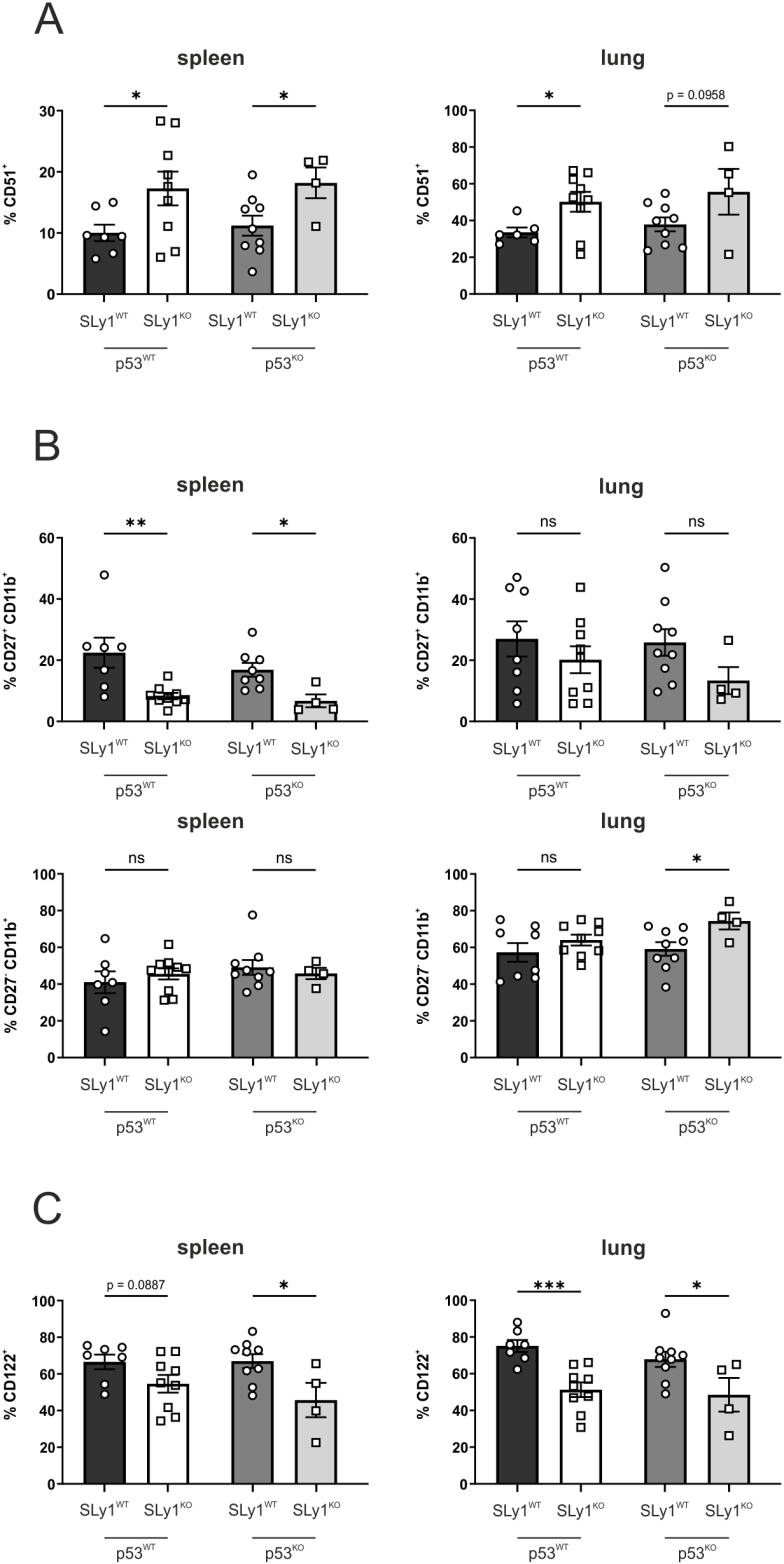
Quantification of different exhaustion markers on the surface of SLy1^KO^ NK cells. Expression of **(A)** CD51, **(B)** CD27^+^ and CD11b^+^ and **(C)** CD122 on NK cells. Error bars represent standard error of the mean. ∗: p <0.05, ∗∗: p <0.01, ∗∗∗: p <0.001, ns, not significant; unpaired t-test.

In the context of T cells, CD51 is considered a possible marker for T cell exhaustion, especially in CD8^+^ T cells (47, 48). The expression of CD51 usually only occurs during the initial stage of mature NK cells and is downregulated during the last steps of NK cell maturation (49, 50). Therefore, we analyzed the percentage of NK cells positive for CD51. Interestingly, in the spleen of SLy1^KO^; p53^WT^ mice the percentage of CD51^+^ NK cells was significantly increased compared to SLy1^WT^; p53^WT^ control animals. Moreover, SLy1^KO^; p53^KO^ mice also showed a higher percentage of CD51^+^ NK cells in the spleen compared to SLy1^WT^; p53^KO^ control animals. In the lungs of SLy1^KO^; p53^WT^ animals, approximately half of the NK cells were positive for CD51, in contrast to one third in SLy1^WT^; p53^WT^ mice. Similar to the spleen, double-deficient SLy1^KO^; p53^KO^ mice also display an increased CD51^+^ NK cell population in the lung, compared to controls. However, this difference is not significant due to fluctuation of the data (**Figure 5A**).

Murine NK cells can be further discriminated into functional differing subgroups based on their expression of CD11b and CD27 (9, 51). CD27^+^CD11b^+^ NK cells are more responsive towards target binding to activating receptors, show increased cytotoxicity after being exposed to different cytokines as IL-12 and IL-18 and interact well with other immune cells. In contrast, CD27^-^CD11b^+^ NK cells are less responsible towards cytokines, produce lower amounts of Interferon-γ and express high levels of Ly49 receptors (9, 51). In the spleen of SLy1^KO^; p53^WT^ mice, the percentage of CD27^+^CD11b^+^ NK cells was significantly decreased compared to SLy1^WT^; p53^WT^ controls. Again, the population could also not be restored to control level in SLy1^KO^; p53^KO^ mice, indicating that the effect is caused by SLy1, independently of p53 (**Figure 5B**, left). In contrast, the CD27^-^CD11b^+^ populations were not drastically altered over all four genotypes (**Figure 5B**, bottom left). In the lungs, we could not detect significant alterations in both population groups, however trends were detectable (**Figure 5B**, right). Interestingly, we could show that the percentages of CD27^-^CD11b^+^ NK cells in the lungs of SLy1^WT^; p53^WT^ specimens are in general higher compared to the spleen, which correlates with previous reported findings (9). In summary, these data show that a SLy1 knockout leads to a reduction in active CD27^+^CD11b^+^ NK cells, which occurs independently of p53.

NK cells are relying on several cytokines for a robust functionality and maturation process. Interleukin-2 and -15 (IL-2 and IL-15) display both indispensable roles for sustaining a proper NK cell development, survival and functionality (52).

CD122 displays the β chain of both the IL-2 and IL-15 receptor and is thus essential to transduce signaling through both interleukins, resulting in expansion and survival of NK cells (53, 54). We could reveal that in the spleen of SLy1^KO^; p53^WT^ mice the frequency of CD122^+^ NK cells was lower in comparison to SLy1^WT^; p53^WT^ controls. In SLy1^KO^; p53^KO^ mice, the additional deletion of p53 could not reverse the population back to control levels of SLy1^WT^; p53^KO^ mice. Similar findings were observed for the lungs, as the CD122^+^ NK cell population in SLy1^KO^; p53^WT^ mice was significantly reduced in comparison to SLy1^WT^; p53^WT^ controls. Again, in double-deficient SLy1^KO^; p53^KO^ mice, the CD122^+^ population was significantly decreased compared to corresponding SLy1^WT^; p53^KO^ mice (**Figure 5C**). It can be assumed that an SLy1 knockout leads to reduced expression of receptors for IL-2 and IL-15.

### Reduced cell count but preserved proliferation capacity in the presence of IL-15 in SLy1^KO^ NK cells

IL-15 is a key mediator in regulating NK cell maturation, survival and proliferation (55–57). Low concentrations support survival whilst high concentrations enhance proliferation and increase cytotoxicity (57). Here, we treated splenocytes for 48 h or 72 h with either a low (10 ng/ml) or a high (100 ng/ml) dosage of IL-15 and analyzed proliferation (*via* KI-67) and frequency of the NK cell population within the heterogenous pool of splenic cells.

**Figure 6:**
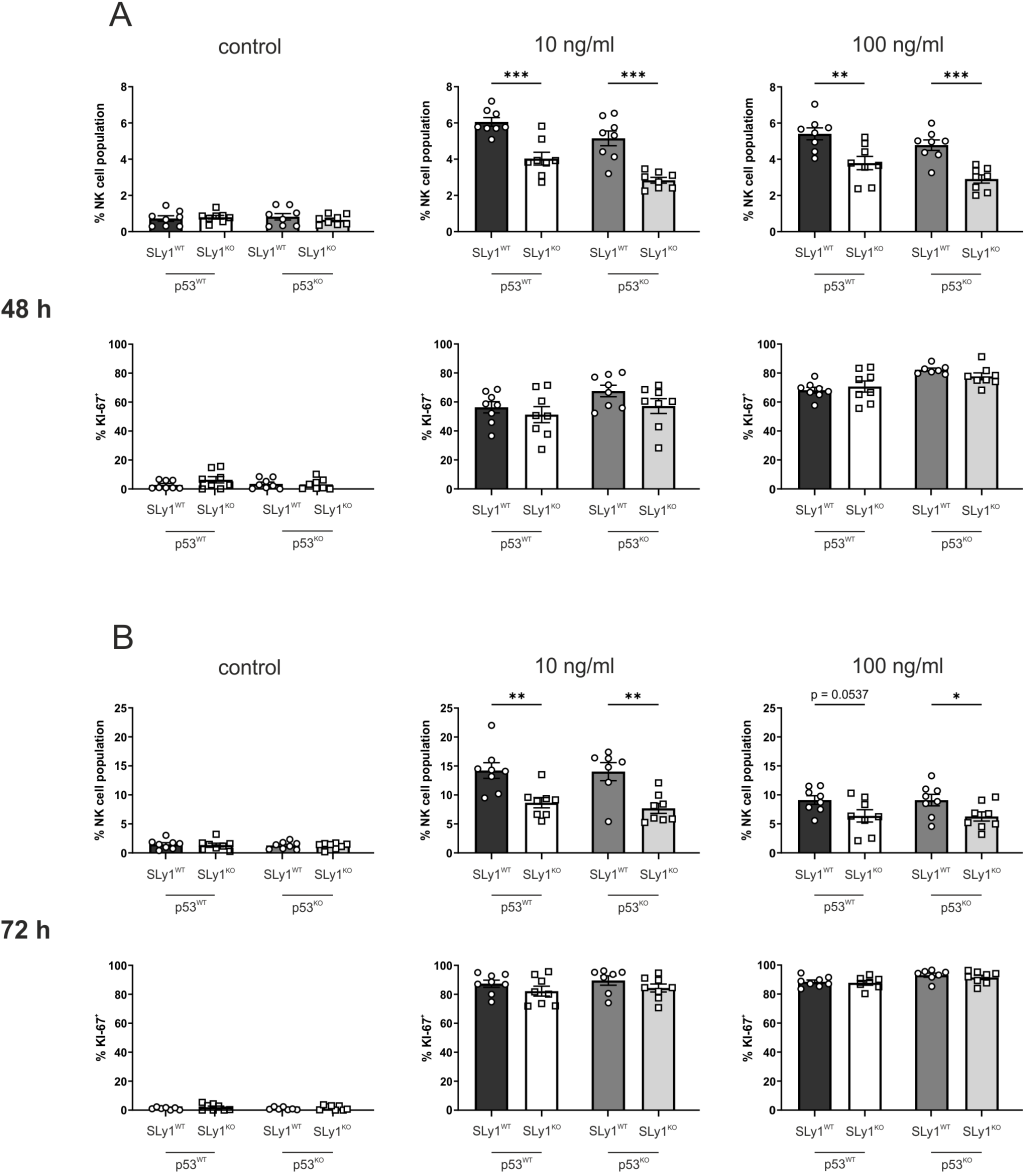
NK cell population and KI-67 expression after *ex vivo* stimulation with IL-15. Freshly isolated splenocytes from mice of the indicated genotypes were cultured with an IL-15 concentration of 10 ng/ml or 100 ng/ml and were analyzed after **(A)** 48 hours and **(B)** 72 hours. Error bars represent standard error of the mean. ∗: p <0.05, ∗∗: p <0.01, ∗∗∗: p <0.0001; unpaired t-test.

After 48 h of incubation, the percentage of NK cells, as well as the frequency of KI-67^+^ NK cells, in the untreated control group was on a similar level between all four genotypes (**Figure 6A**).

Stimulation of splenocytes with a low concentration of IL-15 resulted in an increase of the NK cell population compared to the unstimulated controls. However, the NK cell populations in SLy1^KO^; p53^WT^ and SLy1^KO^; p53^KO^ specimens were significantly smaller as in the corresponding control groups. Nonetheless, we could not detect differences in the proliferation capacity, displayed by the percentage of KI67^+^ NK cells, between all genotypes.

Similar findings were made for the stimulation with a high concentration (c = 100 ng/ml) of IL-15: whilst the stimulation leads to an increase of the NK cell population in general, the NK cell populations of SLy1^KO^; p53^WT^ and SLy1^KO^; p53^KO^ specimens were significantly reduced compared to controls. Again, the percentage of KI-67^+^ NK cells was not altered between the four genotypes.

After 72 h of incubation, we could detect similar findings as after 48 h (**Figure 6B**). Both concentrations of IL-15 led to an increase of the size of the NK cell population, however the NK cell populations of SLy1^KO^; p53^WT^ and SLy1^KO^; p53^KO^ genotypes remained smaller than their controls. Again, we could not detect differences in the frequency of KI-67^+^ NK cells.

These findings indicate that SLy1^KO^; p53^WT^ and SLy1^KO^; p53^KO^ NK cells are in general responsive towards IL-15 and their proliferation capacity depending on IL-15 is not impaired. Still, the unchanged proliferation activity is not sufficient to overcome the difference in population size between SLy1^WT^; p53^WT^ and SLy1^KO^; p53^WT^ as well as SLy1^WT^; p53^KO^ and SLy1^KO^; p53^KO^, which could possibly be explained by the reduced expression of IL-15 receptors.

### SLy1-deficiency leads to altered expression of genes and proteins associated with DNA damage response

The above presented results could show that an additional deletion of p53 in SLy1^KO^ NK cells can restore several phenotypic alterations back to control levels. To gain more understanding about the interplay between SLy1 and p53, we performed a p53 signaling pathway array, in which 84 genes involved in the p53 signaling cascade were screened regarding potential changes in their expression levels. The isolated RNA was transcribed into cDNA and after a pre-amplification, a RT^2^ Profiler PCR Array was performed and analyzed with the Qiagen GeneGlobe tool.

We could identify seven dysregulated genes in the p53 array in splenic SLy1^KO^ NK cells, compared to SLy1^WT^ controls. *Xrcc4* and *Zmat3* were shown to be downregulated in SLy1^KO^ NK cells, whereas *Atr, Chek1* and *Sirt1* displayed an increased mRNA level (**Supplementary Figure 4**). *Serpinb5* and *Nf1* were also found to be upregulated, but the average threshold cycles were >30 cycles. This indicates that their relative expression level was very low, making it likely that the values were false-positive results and were therefore not pursued further.

**Figure 7:**
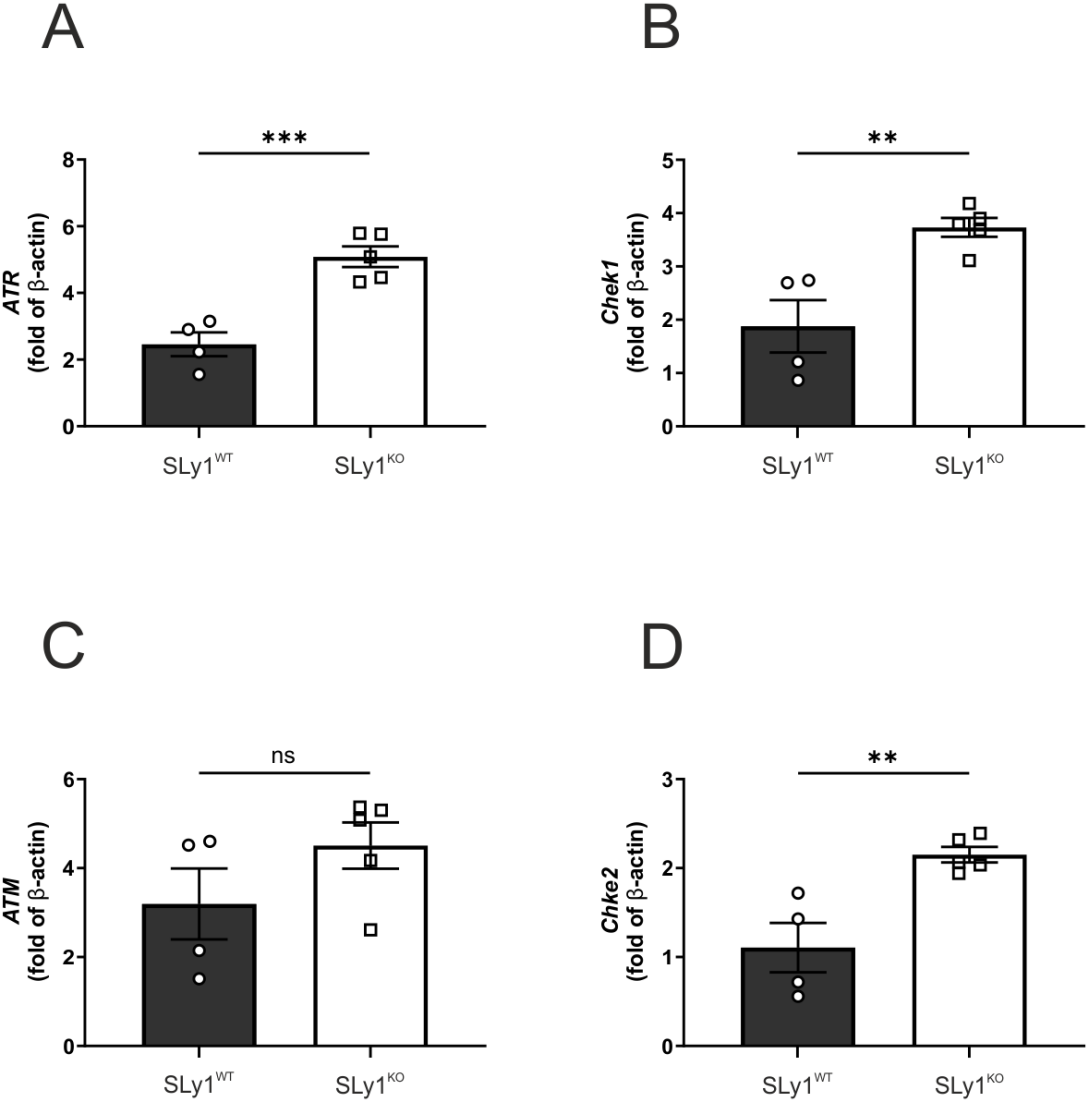
The mRNA expression was quantified in NK cells by qPCR. Figures show the expression in relation to β-actin of **(A)** *Atr*, **(B)** *Chek1*, **(C)** *Atm* and **(D)** *Chek2*. Error bars represent standard error of the mean. ∗∗: p <0.01, ∗∗∗: p <0.001, ns = not significant; unpaired t-test.

After obtaining a rough overview using the p53 signaling pathway array, we performed a more detailed quantification of individual genes using qPCR. We focused on further analysis of *Atr* and *Chek1* and also added *Atm* and *Chek2*, as these genes are closely linked to each other as members of the DNA damage response. Quantification *via* qPCR revealed a statistically significant upregulation of mRNA expression levels of *Atr, Chek1* and *Chek2* in SLy1^KO^ NK cells compared to wildtype controls. Concerning the expression of *Atm*, the results showed a tendency towards an upregulation in SLy1^KO^ NK cells, but the values scattered too widely for statistical significance (**Figure 7**).

**Figure 8:**
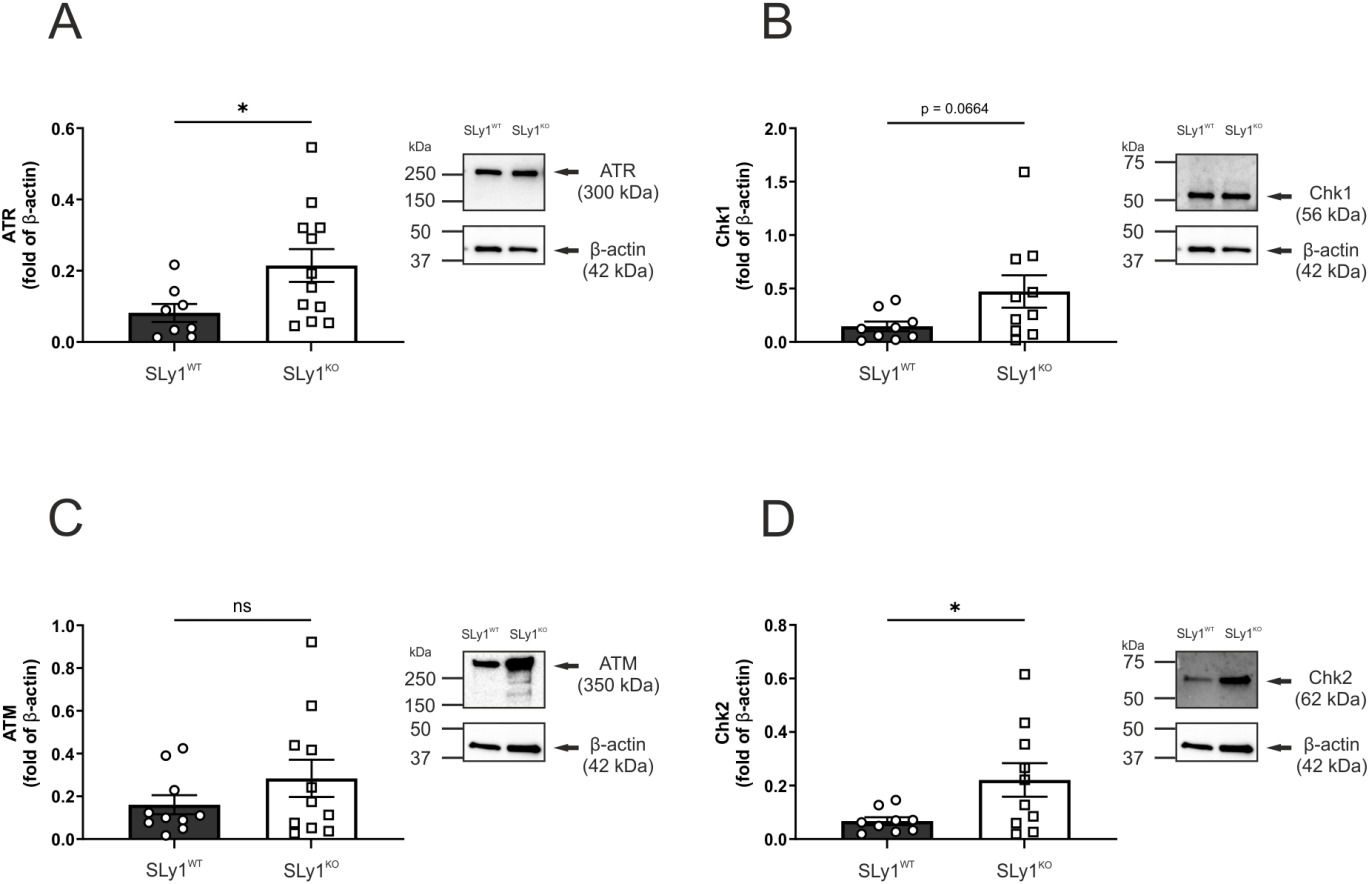
The protein expression was quantified in NK cells by immunoblotting. Figures show representative blots and analysis of the expression in relation to β-actin of **(A)** ATR, **(B)** Chk1, **(C)** ATM and **(D)** Chk2. Error bars represent standard error of the mean. ∗: p <0.05, ns, not significant; unpaired t-test.

We aimed to understand whether SLy1^KO^ NK cells also display an elevation of the DDR mediators on protein level. Therefore, we quantified the four proteins using conventional immunoblotting and calculated the expression level in ratio to ß-actin. We could reveal that all four proteins are elevated in SLy1^KO^ NK cells compared to SLy1^WT^ controls, with ATR and Chk2 showing a significant elevation (**Figure 8**).

We could show here for the first time that the absence of SLy1 leads to an upregulation of mediators of the DDR pathway in NK cells, both on protein and mRNA level.

## Discussion

As in all other cell types, the transduction of extracellular signals through an intracellular signaling cascade is essential for lymphocytes. Adapter proteins play a crucial role in this process. Though they do not display an enzymatic activity, their specific domains enable protein-protein interactions and can therefore enable the transfer of extracellular signals to an intracellular level (58). SLy1 is an emerging representative of the adapter protein family, playing an important role in lymphocyte function, viability and activation (12). Current case studies emphasize the importance of the presence of intact *SLy1* alleles, as there are several patients reported displaying mutations in the *SLy1/SASH3* gene locus. These patients exhibit an inborn X-linked common variable immunodeficiency and show symptoms of recurring infections, reduced numbers of lymphocytes and signs of autoimmunity. Whilst symptoms often already appear during childhood, it usually takes several years until a pathogenic genetic variant is diagnosed (17–19). Standard gene sequencing panels for immunodeficiencies do not yet include screening for *SLy1/SASH3*. Probably, because its function in human and murine models has not completely been elucidated, although mutations within the gene locus can have severe outcomes.

Studies in mice have shown that SLy1 plays a crucial role in lymphocyte survival, functionality and maturation. SLy1^KO^ mice display substantial reductions in the size of lymphoid organs, such as the spleen, caused by drastic reductions of NK, T and B-cell numbers. In murine T and B lymphocytes, SLy1 has the ability to shuttle between cytoplasm and cell nucleus (12). However, as previously shown by our research group and others, in murine NK cells SLy1 is exclusively located in the cytoplasm, where it serves as ribosomal stabilizer (13). In the absence of SLy1, ribosomal instability occurs in NK cells, leading to the formation of free ribosomal proteins (13). This SLy1-mediated ribosomopathy leads to a dysfunction of SLy1^KO^ NK cells, displayed by a reduced NK cell count, inefficient degranulation and decreased cytotoxicity towards target cells (13). Interestingly, the deficiency of SLy1 has a more severe outcome in splenic NK cells in contrast to lung NK cells, which seem to be less affected regarding apoptosis and the expression of activating receptors. This is congruent with other studies reporting that NK cells in lung and spleen are phenotypically distinct, whilst lung NK cells show a higher degree of maturation compared to those in the spleen (10, 11, 59, 60). These findings are also consistent with other ribosomopathies with known mutated ribosomal or ribosome-associated proteins, which often exhibit tissue specificity (61). A possible hypothesis could be the fact that tissue-resident lung NK cells are exposed to a different environment (e.g. more interleukins, chemokines, stimulation by antigens) (62).

Ribosomal dysfunction, similar to impaired ribosome biogenesis, can lead to nucleolar stress response (also known as impaired ribosome biogenesis checkpoint), which induces activation of the p53 signaling pathway, resulting in cell cycle arrest, senescence, and apoptosis (63, 64).

**Figure 9:**
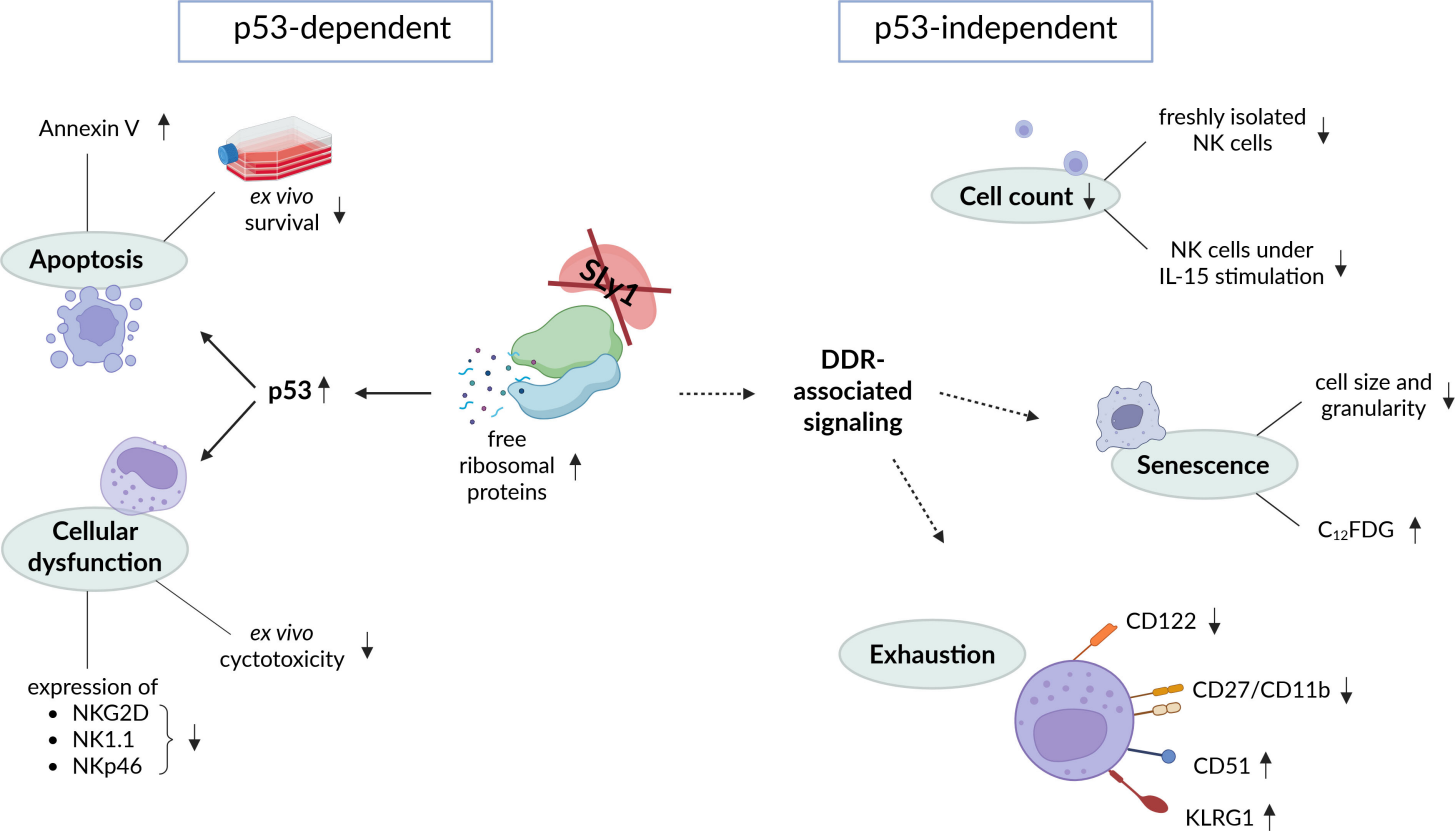
Schematic presentation of the effect of SLy1-deficiency on the phenotype and function of NK cells and the relationship to p53. Increase in free ribosomal proteins was previously shown by *Arefanian et al.* Dashed lines represent hypotheses. Created in BioRender. Beer-Hammer, S. (https://BioRender.com/hjgbedc) is licensed under CC BY 4.0.

p53 is known as a key player for maturation, functionality and cell cycle progression of NK cells (65, 66). Here, we were able to show for the first time the crucial role of p53 in several phenotypic alterations in SLy1-deficient NK cells, as the additional (NK cell specific) deletion of p53 could restore the deficiencies back to control levels. These include apoptosis, *ex vivo* survival, the expression of activating surface receptors and the *ex vivo* cytotoxicity towards cancer cells (**Figure 9**, left).

In the context of the present study, we were able to reproduce the unfavorable effect of a SLy1^KO^ on NK cell viability, described by A*refanian et al.* as we also observed an increase in Annexin V^+^ NK cells in SLy1^KO^ mice. Further, SLy1^KO^ NK cells displayed significantly lower survival rates under culture conditions in *ex vivo* experiments. The cellular dysfunction of splenic SLy1^KO^ NK which we observed, is characterized by decreased expression levels of activating NK cells markers and a decreased cytotoxicity against lung cancer cells *ex vivo*.

Several other studies focus on how the expression of p53 in target cells can shape the expression of ligands for activating NK cell surface receptors (67–69). Here, we could demonstrate that SLy1, together with p53, is crucial for a physiological expression of activating NK cell receptors and in turn a proper cytotoxicity against malignant cells. *Arefanian* et al. already revealed that SLy1-deficient NK cells displayed severe deficits in lysing LLC cells and postulated that this dysfunction occurs in a p53-dependent manner (13). We were able to confirm these results and demonstrate for the first time that this defect truly arises in dependence on p53, as the absence of both proteins leads to a restoration of the cytolytic function.

However, we could also identify phenotypic alterations in SLy1^KO^ NK cells that apparently arise p53-independently (**Figure 9**, right). These are in particular a tendency towards an increased senescence and exhaustion, as well as a reduction in NK cell count, in both the absence or presence of IL-15. We could reveal that SLy1^KO^ NK cells display divergent surface levels of several receptors, representing an exhausted phenotype. In detail, these affect the expression of CD122, CD27/CD11b, CD51 and KLRG1 and occur in a p53-dispensible manner, indicating that SLy1 alone is causative for the altered receptor levels. Additionally, the reduced cell size and granularity, combined with an increased C_12_FDG uptake strongly indicate a trend to a senescent state in SLy1^KO^ NK cells, independent from p53. Studies in cancer cells could show that a derailed ribosomal RNA synthesis is connected to cancer cell proliferation (70, 71). By inhibiting this synthesis pathway in solid cancer cell lines, it was possible to induce senescence and autophagy in a p53-independent manner (72). This emphasizes the tight connection between intact ribosomes and senescence and could provide an underlying mechanism for the observations described in this work. Likewise, senescence and exhaustion can both be initiated by an activation of the DDR signaling cascade (43, 73). In SLy1^KO^ NK cells we observed an upregulation of mediators of the DDR signaling cascade at both mRNA and protein levels, which could be involved in the effects described above.

It is reported that ribosomal proteins have the ability to induce the DDR signaling cascade in a p53-dependent and -independent manner (61). Interestingly, there is no need for DNA damage but impairments in the ribosomal biogenesis, ribosomal stress or free ribosomal proteins are capable of triggering the signaling pathway (61, 74, 75). The absence of SLy1 itself does not cause DNA damage but probably provokes the DDR signaling cascade through a so far unknown mechanism. We therefore propose that the upregulated mediators of the DNA damage response in SLy1^KO^ NK cells could be causative for reduced cell numbers, exhaustion and senescence in a p53-independent manner, as the additional knockout of p53 does not lead to a restoration of the phenotypical alterations to control level.

Interestingly, several studies also report a tight connection between IL-15 and the DDR, as the administration of IL-15 can reduce the expression levels of mediators involved in the DDR in several different cell types (76, 77). We were able to show that IL-15 triggers proliferation in SLy1^KO^ NK cells in the same frequency as controls. However, the equal proliferation capacity is not sufficient to overcome the differences in the size of the NK cell populations. As the basal number of NK cells is already reduced in SLy1^KO^; p53^WT^ and SLy1^KO^; p53^KO^ specimens, the frequency of proliferating cells would need to be higher to overcome the initial decrease in the NK cell count. Interestingly, *Arefanian et al.* showed previously that the treatment of SLy1^KO^ NK cells with IL-2 resulted in the restorage of several phenotypical alterations, such as the clearance of tumor cells and the expression of activation markers (13). IL-2 and IL-15 both signal through the same receptor complex, therefore it is possible that IL-15 could have similar effects on SLy1^KO^ NK cells. These results are supported by the finding that proliferation rates converge after stimulation, even with a low dosage of IL-15. This is particularly surprising, as we detected decreased levels of CD122 on SLy1^KO^; p53^WT^ NK cells, which displays the β-subunit of the IL-2/IL-15 receptor and is therefore crucial for sustaining the IL-15 signaling. Signaling through IL-15 results in the activation of several different pathways, among them mTOR signaling, leading to NK cell growth, increased uptake of nutrients, proliferation and the production of IFN-y (78–80). Studies report that the inhibition of mTOR can result in cell cycle arrest and apoptosis in a p53-independent manner (81). Data from our group could reveal that SLy1^KO^ thymocytes show impairments in activating the mTOR signaling complex (20). It is possible that these impairments also occur in SLy1^KO^ NK cells and therefore the treatment with a comparable high dosage of IL-15 helps to overcome those deficiencies.

Defects in ribosomal biogenesis, translation, and the functions of individual ribosomal proteins have been linked to a variety of congenital diseases and higher risk of developing cancer (82, 83). Therefore, it can be assumed that stable ribosomes are crucial for cell function and that ribosomal dysfunction in NK cells, for example due to a mutation of the *SLy1* gene, could lead to similar, possibly milder symptoms. We strongly recommend considering screenings for mutations in the *SASH3/SLy1* locus for patients with unclear clinical symptoms, especially in cases associated with immunodeficiency. The inclusion of the SLy1 gene in screening panels for primary immunodeficiencies should be discussed.

## Data Availability

The original contributions presented in the study are included in the article/**Supplementary Material**. Further inquiries can be directed to the corresponding author.
