# Supplementary material for: SLy1-deficiency results in functional impaired, exhausted and senescent NK cells
Source: Front Immunol. 2026 Jun 4;17:1836862. doi: 10.3389/fimmu.2026.1836862 (PMC13275445; doi:10.3389/fimmu.2026.1836862)
Supplement: Supplementary file 1 [file Table1.docx]

# Supplementary data

**Table S1.** Mouse genotypes and their abbreviations used in this study.

| Genotype | Referred as |
| --- | --- |
| SLy1^WT^; p53^flox/flox^; Ncr1-Cre^WT^ | SLy1^WT^; p53^WT^ |
| SLy1^KO^; p53^flox/flox^; Ncr1-Cre^WT^ | SLy1^WT^; p53^KO^ |
| SLy1^WT^; p53^flox/flox^; Ncr1-Cre^tg^ | SLy1^WT^; p53^KO^ |
| SLy1^KO^; p53^flox/flox^; Ncr1-Cre^tg^ | SLy1^KO^; p53^KO^ |

**Table S2**. Target gene sequence of the specific primers used in this study.

| Target | Sequence (5’→3’) |
| --- | --- |
| *Atm fwd* | tgcagatttatatccatcatccac |
| *Atm rev* | ttcatggattcataagcacctt |
| *Atr fwd* | tggagagtcacgacttgctg |
| *Atr rev* | aacaataagcgcctggtgaa |
| *Chek1 fwd* | gagggaaggccatatccagt |
| *Chek1 rev* | ttgttcaggcatccctatgtc |
| *Chek2 fwd* | ttattcctgaagtctggacagatg |
| *Chek2 rev* | ctaacagtttcttgacaaggtcca |


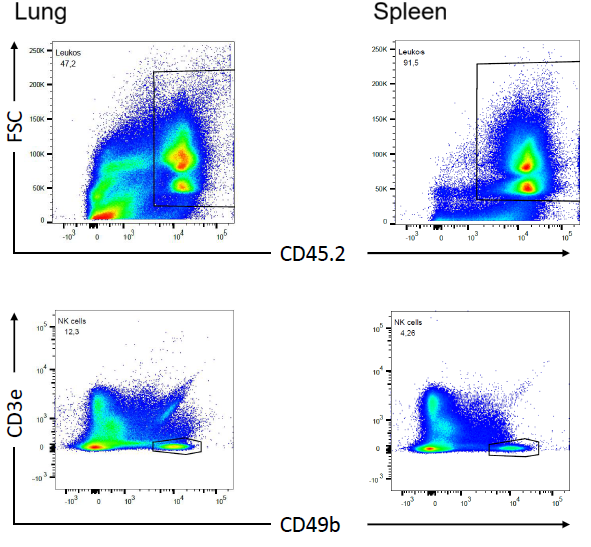


**Figure S1.** Gating strategy for NK cells in cell suspension from lung and spleen. NK cells were defined as CD45.2^+^ CD3e^-^ CD49b^+^.


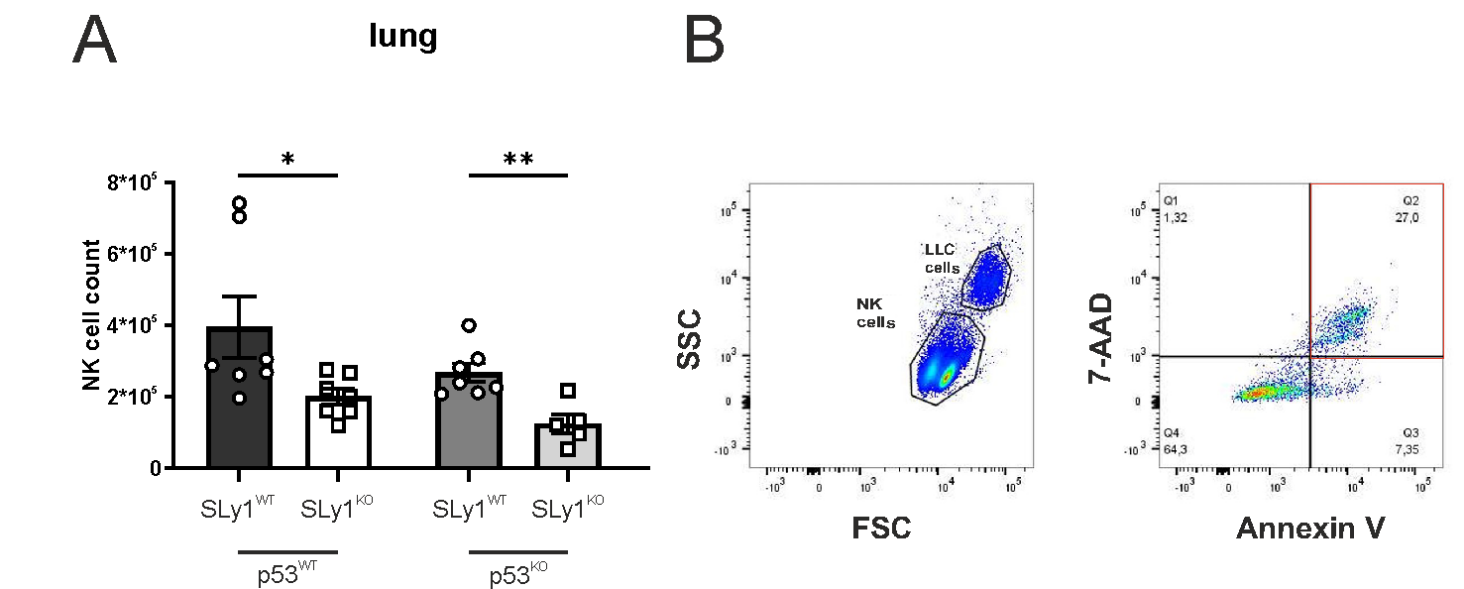


F**igure S2.** **(A)** Phenotypic effects of a SLy1 knockout on pulmonary NK cells in dependence on p53. Absolute NK cell count determined *via* flow cytometry in the lungs of mice of the specified genotypes. error bars represent standard error of the mean. ∗: p <0.05, ∗∗: p <0.01; unpaired t-test. **(B)** Representative gating plot for NK cell cytotoxicity assay. NK cells and LLC cells were discriminated based on their FSC/SSC values. LLC cells were then further distinguished between living and dead cells based on their Annexin V and 7-AAD signal (red rectangle).


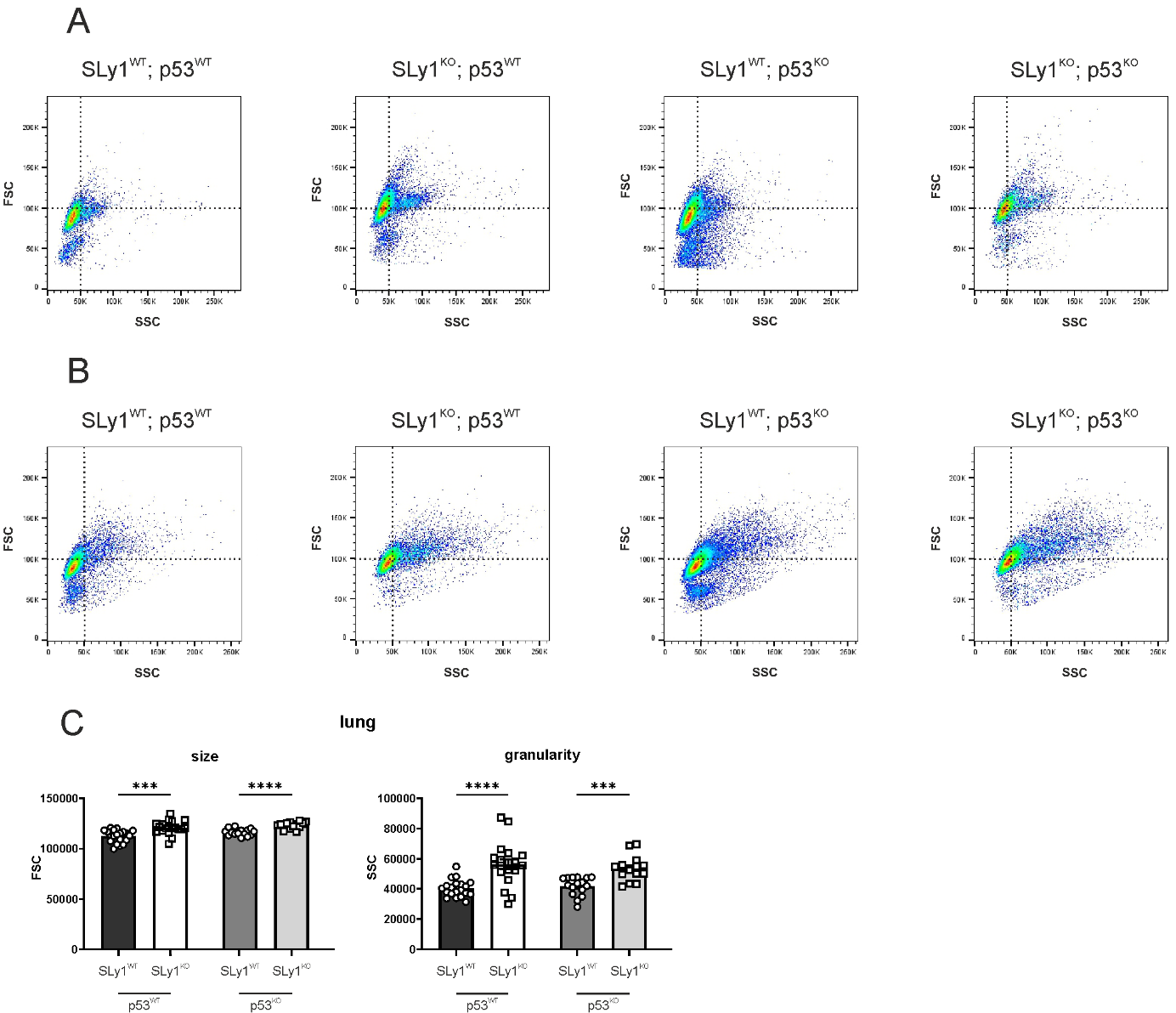


**Figure S3.** Representative FSC/SSC dot plots of **(A)** splenic and **(B)** lung SLy1^WT/KO^; p53^WT/KO^ NK cells. Pre-defined CD45.2^+^ CD3e^-^ CD49b^+^ NK cells were again plotted for FSC vs. SSC. **(C)** Analysis of FSC and SSC in pulmonary NK cells with large sample size. In all figures error bars represent standard error of the mean. ∗: p <0.05, ∗∗: p <0.01, ∗∗∗: p <0.001, ∗∗∗∗: p <0.0001; unpaired t-test.


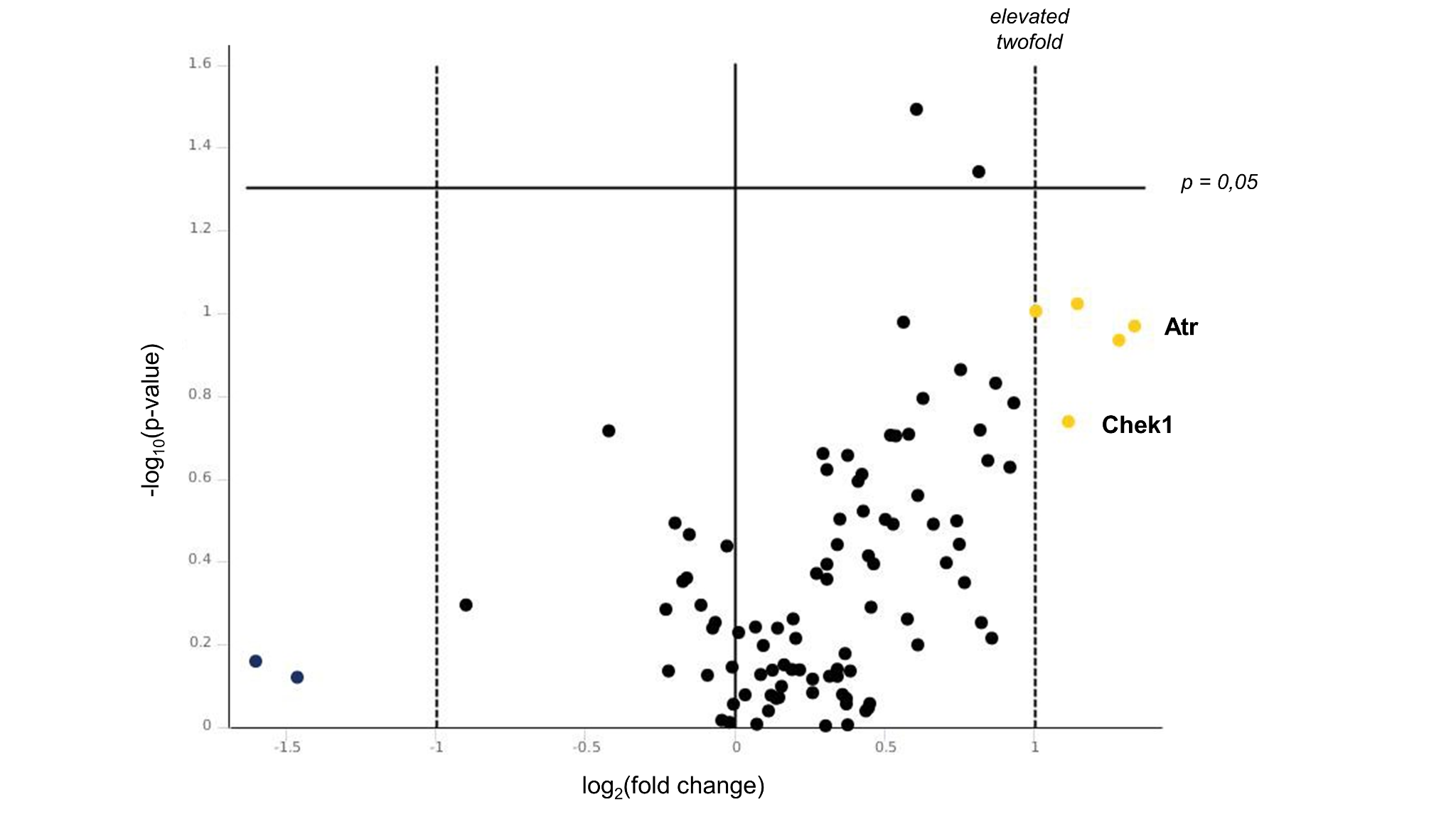


**Figure S4.** Analysis of dysregulated genes in SLy1^KO^ NK cells within the p53 signaling network. The volcano plot shows statistical significance (y-axis) versus fold change (x-axis), combining a statistical p-value test with fold change to quickly identify genes with expression changes.
